# Mode of delivery, perinatal outcome and neurodevelopment in uncomplicated monochorionic diamniotic twins: a single-center retrospective cohort study

**DOI:** 10.1186/s12884-022-04425-4

**Published:** 2022-02-01

**Authors:** Angel Chimenea, Lutgardo García-Díaz, Guillermo Antiñolo

**Affiliations:** 1 Department of Materno-Fetal Medicine, Genetics and Reproduction, Institute of Biomedicine of Seville (IBIS), Hospital Universitario Virgen del Rocio/CSIC/University of Seville, Seville, Spain; 2 Fetal, IVF and Reproduction Simulation Training Centre (FIRST), Seville, Spain; 3 Department of Surgery, University of Seville, Seville, Spain; 4 Centre for Biomedical Network Research On Rare Diseases (CIBERER), Seville, Spain

**Keywords:** Twin birth, Planned vaginal delivery, Mode of delivery, Monochorionic twin

## Abstract

**Background:**

There is no agreement on the effect of planned mode of delivery in the perinatal morbidity and neurodevelopment in uncomplicated monochorionic diamniotic as well as regarding the safest mode of delivery. In this paper we have aimed to analyze the impact of the mode of delivery in uncomplicated monochorionic diamniotic twins ≥ 32 weeks of gestation.

**Material and methods:**

This study included 72 women, followed and attended at our department, with uncomplicated monochorionic diamniotic pregnancies who had a birth between 32.0 and 37.6 weeks of gestation from January 2012 to December 2018. Outcomes were recorded in women who underwent planned vaginal delivery (induced or spontaneous onset of labor), and women who underwent a planned cesarean section for any reason that excluded vaginal delivery. Primary outcomes included: (1) A composite of any of the following: neonatal death, 5-min Apgar score < 4, respiratory distress syndrome, bronchopulmonary dysplasia, sepsis, periventricular leukomalacia, intraventricular hemorrhage, and necrotizing enterocolitis. (2) Neurodevelopmental status at 2 years of corrected age.

**Results:**

In this period, 42 women (58.3%) had a planned vaginal delivery, and 30 women (41.7%) had a planned cesarean section. In the first group, 64.3% had a vaginal delivery. The rate of successful vaginal delivery was similar regardless the onset of labor. We did not find a higher composite perinatal morbidity in the planned vaginal delivery group (planned vaginal delivery: 3.6% vs. planned cesarean section: 8.3%, aOR 1.36, 95% CI 0.24–7.81). Considering the onset of labor, it was more frequent in the spontaneous subgroup (8.3% vs. 0%). The rate of neurodevelopmental impairment was higher in the planned cesarean section group, without reaching statistical significance [10.2% vs. 4.9%, aOR 1.53 (95% CI 0.37–6.29)].

**Conclusions:**

In uncomplicated monochorionic diamniotic twins at ≥ 32 weeks of gestation, when the first twin is in vertex presentation, our results suggest that planned vaginal delivery is safe, with a successful outcome as well as high vaginal delivery rate.

## Background

Compared to singleton pregnancies, twin pregnancies are at a higher risk for an adverse perinatal outcome [1–3]. Hence, the optimal mode of delivery is a particularly contentious issue. A long-lasting debate keeps the controversy going; while some authors suggest that vaginal delivery is a safe option for uncomplicated twin pregnancies [4–7], other investigators recommend planned caesarean section (PCS) in order to avoid intrapartum complications, especially those derived from breech extraction [8–10].

The dilemma in monochorionic diamniotic (MCDA) twins is greater, as vascular anastomoses between fetal circulations may result in acute and dramatic hemodynamic intrapartum changes [11–13]. Even in uneventful monochorionic pregnancies, likelihood of adverse perinatal outcomes is higher compared with singletons and dichorionic twin pregnancies [11, 14, 15].

Although chorionicity has a significant influence on perinatal outcomes, most studies dealing with the mode of delivery in twins do not stratify this topic accordingly [5, 16]. Furthermore, published studies regarding the mode of delivery in MCDA pregnancies do not consider essential outcome issues as induction of labor nor neurodevelopment status [17–24]. In addition, the results in most reports are based on national databases analysis, which makes data difficult to extrapolate, as far as there are no common and homogeneous obstetric policies. Furthermore, neonatal criteria for defining morbidity are different, and in many cases do not cover all the main complications of MCDA pregnancies.

The purpose of our study has been to analyze perinatal outcomes and 2-year-neurodevelopment status for moderately to late preterm and early term MCDA twins with respect to the mode of delivery, as well as to assess the safety of the induction of labor.

## Material and methods

### Study design, setting and population

This is a retrospective cohort study conducted at Virgen del Rocío University Hospital of Seville (a tertiary referral center with an average of 6000 births per year), including all uncomplicated MCDA pregnancies who had delivery at 32.0 – 37.6 weeks of gestation from 2012 to 2018. All the pregnancies were followed and assisted in our center, as well as subsequent follow-up up to two years of life, by a homogeneous follow-up protocol throughout the entire study period.

Management protocols for MCDA twins were determined locally following the criteria derived from international guidelines, taking into account their updates during the study period, as those established by the Royal College of Obstetricians and Gynaecologists [25, 26], or the International Society of Ultrasound in Obstetrics and Gynecology [26].

Chorionicity was determined by first-trimester ultrasonography and confirmed after birth by pathological examination. In all cases a detailed anomaly scan was performed at 18.0 – 21.6 weeks. Fetal ultrasound assessment was performed every 2 weeks from 16.0 weeks onwards until delivery. At every ultrasound examination, liquor volume in each of the amniotic sacs, estimated fetal weight, visualization of fetal bladders, middle cerebral artery peak systolic velocity value, as well as the umbilical artery pulsatility index of both fetuses were recorded. In uncomplicated pregnancies, induction of labor is considered from 37.0 to 37.6 weeks, unless other clinical indications for caesarean section. Induction is undertaken using vaginal prostaglandin E2 delivery system. The delivery was attended by at least two senior obstetricians and two neonatologists. Neurodevelopmental assessment was performed in a specific monographic consultation, at least three times in the first two years of life (2, 12 and 24 months of life).

Cases were classified retrospectively by the authors into induced or spontaneous onset of labor (planned vaginal delivery, PVD) or PCS groups depending on delivery method. PVD was considered after 32 weeks of gestation, when there was no contraindication for vaginal delivery (non-vertex first twin, at least two previous CS, weight discordance > 15% when first twin was smaller, vasa previa, and/or any other condition like active herpes genital infection), if twin A was in vertex presentation, and estimated fetal weight was at least 1500 g.

In non-vertex presentation of the second twin, breech extraction was performed when gestational age at delivery was 32 weeks or greater, in the absence of a previous CS, and intertwin weight discordance was less than 15%. PCS was not performed because of maternal request.

### Outcomes

We have established a composite of perinatal morbidity as primary outcome, with any of the following: neonatal death, 5-min Apgar score of < 4, respiratory distress syndrome (RDS), bronchopulmonary dysplasia (BPD), sepsis, periventricular leukomalacia (PVL), intraventricular hemorrhage (IVH) and necrotizing enterocolitis (NE). 5-min Apgar score of < 7 was registered as well. Like other authors [24], we did not include umbilical cord pH at birth as an outcome, as we found a significant interaction with 5-min Apgar score.

We also analyzed the neurodevelopment status at 2 years of corrected age. For this purpose, we used the revised Brunet-Lézine scale [27]. Infants were classified as having neurodevelopmental impairment (NDI) if they had a global developmental quotient (DQ) on the revised Brunet–Lézine scale of < 85 or level 3 cerebral palsy, and severe NDI was defined with a DQ of < 60.

### Enrolment criteria

We successively enrolled all pregnant women with MCDA twins that met the criteria summarized in Table 1. We only included MCDA twin pregnancies when both fetuses were alive after 32 weeks of gestation.

**Table 1:** Enrolment criteria

Inclusion criteria	Exclusion criteria
**PVD between 32.0 and 37.6 weeks**	Stillbirth Congenital anomaly Severe preeclampsia Antepartum cardiotocography pathology Severe IUGR Selective IUGR Twin-to-twin transfusion syndrome Twin anemia-polycytemia sequence Gestational age less than 32 weeks or greater than 38 at the time of delivery Uncertainty about the gestational age at birth
• Spontaneous onset of labor (between 32.0–37.6 weeks) or induced onset of labor (between 35.0–37.6 weeks) in uncomplicated MCDA twin pregnancy with no contraindication for vaginal delivery	
**PCS between 32.0 and 37.6 weeks**	
• Spontaneous onset of labor in uncomplicated MCDA twin pregnancy with contraindication for vaginal delivery (non-vertex first twin, at least two previous CS, weight discordance > 15% when first twin was smaller, vasa previa, estimated fetal weight of twin A < 1500 g and/or any other condition like active herpes genital infection) • In non-vertex presentation of the second twin: gestational age at delivery < 32.0, previous CS, and/or intertwin weight discordance > 15%	
PCS regarding to: ° Previous CS (in induced onset of labor) ° Positional placental anomaly ° First twin in non-vertex presentation ° Another obstetric non-urgent indication for PCS	

*PVD* planned vaginal delivery, *MCDA* monochorionic diamniotic, *PCS* planned cesarean section, *CS* cesarean section, *IUGR* intrauterine growth restriction

### Exposures

In our study, prior to labor, pregnant women in which attempted vaginal delivery was clinically allowed were selected and classified as the exposed group (PVD group). Following this, mode of delivery was categorized as PCS or PVD, including spontaneous vaginal vertex deliveries, assisted vaginal birth, breech extraction of the second twin, and emergent cesarean section.

In case of induction of labor, breech extraction, or PCS, an informed consent detailing the risks and benefits of each procedure was explained and signed.

### Data source

Data were extracted from the electronic health record. Later, anonymized data analysis was provided by a randomly generated study identifier.

### Statistical analysis

Statistical analysis was performed using the SPSS 25.0 software package (SPSS Inc., Chicago, IL), and statistical significance was assumed at *P* < 0.05. All hypothesis tests were two-sided.

Values were expressed as mean ± standard deviation (SD) since data were normally distributed, and categorical variables were expressed as numbers and percentages. Univariable comparisons of categorical variables were performed using a chi‐squared test or Fisher's exact test. Comparison of normally distributed continuous variables was performed using Student’s T-test, and non-normally distributed variables with the Mann–Whitney U-test. Binary logistic regression models were used to calculate odds ratios (OR), adjusted odds ratios (aOR) and 95% confidence intervals (CI). In order to control for major confounders, OR was adjusted for birth weight and gestational age at birth.

### Ethical approval

Institutional Review Board approval of this study was obtained from the Andalusian Ethical Committee (Spain) on 11 October 2019 (ref. Nº 1318-N-19). The requirement for informed consent was waived because the data were de-identified. The research protocol was submitted to the Andalusian Ethical Committee before starting the collection of data.

## Results

### Participants characteristics

Over that 6-year period, there were 84 MCDA twin deliveries greater than 32 weeks of gestation of pregnancies who were fully followed-up in our department.

Of those, cases with twin-to-twin transfusion syndrome (TTTS) (*n* = 5), stillbirth (*n* = 3), severe preeclampsia (*n* = 1), severe intrauterine growth restriction (IUGR) (*n* = 2), and gestational age at birth < 32 weeks (*n* = 5) were excluded from the study (some of the pregnancies shared more than one of the above criteria).

**Fig. 1:**
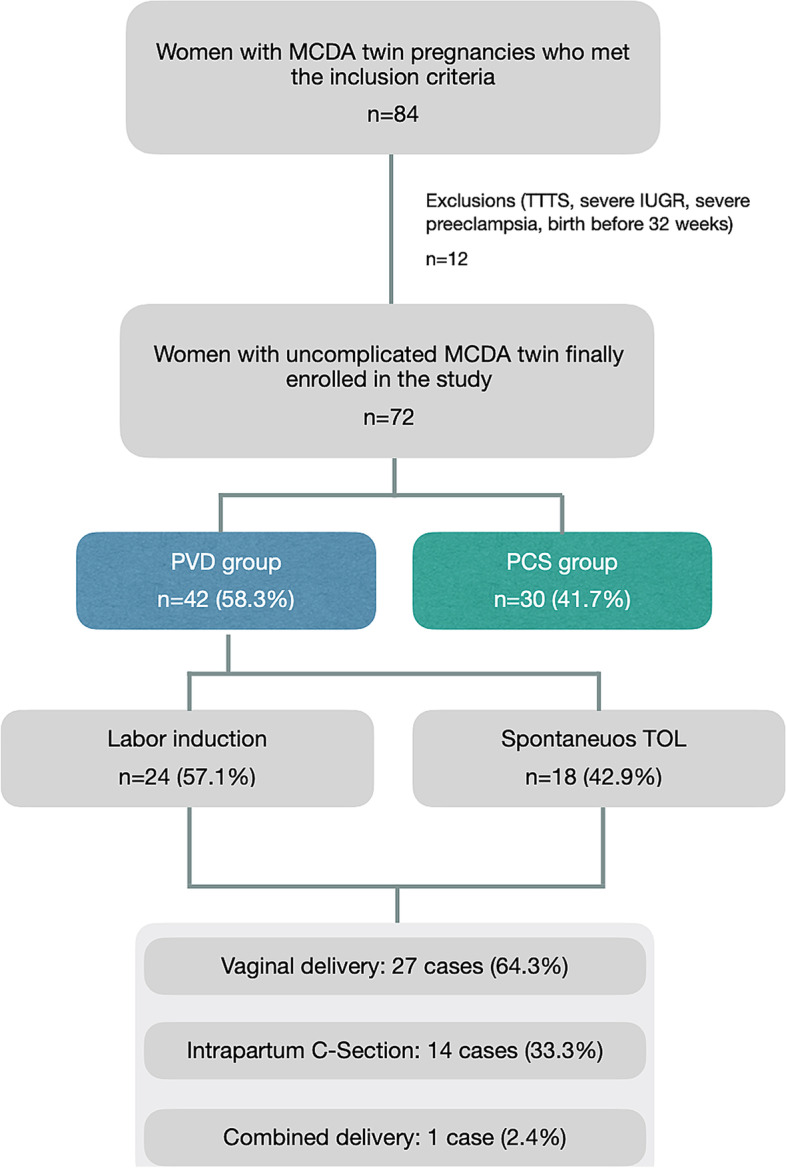
Flow chart of participant enrolment

The final analysis included 72 uncomplicated MCDA twin pregnancies: 42 women (58.3%) in the PVD group and 30 women (41.7%) in the PCS group. The flow chart of participant enrolment is shown in Fig. 1.

Demographic data, clinical features and neonatal characteristics are summarized in Table 2. No statistically significant differences were found regarding mean gestational age at birth (PVD: 36.2 weeks vs. PCS: 35.3 weeks, *P* = 0.15). The mean weight at birth was lower in the PCS group, both for the first twin (2,228 g vs. 2,413 g, *P* = 0.07) and the second one (2,134 g vs. 2,385 g, *P* = 0.02).

**Table 2:** Maternal baseline characteristics and delivery data in PVD and PCS groups

Variable	PVD group *n* = 42 (58.3%)	PCS group *n* = 30 (41.7%)	*P*-value
Maternal age, years	40 ± 4.96	40 ± 6.23	0.83
Maternal weight, kilograms	65.9 ± 16.77	63.4 ± 12.84	0.78
Body Mass Index (BMI), kg/m^2^	23.9 ± 5.22	24.5 ± 5.14	0.44
Previous CS, n(%)	1 (2.4%)	10 (33.3%)	** < 0.001**
Previous vaginal birth, n(%)	18 (42.9%)	8 (26.7%)	0.16
IVF pregnancy, n(%)	8 (19.0%)	6 (20.0%)	0.92
Pregestational diabetes, n(%)	0 (0%)	1 (3.3%)	0.42
Gestational diabetes, n(%)	3 (7.1%)	1 (3.3%)	0.49
Smoking habit, n(%)	11 (26.2%)	8 (26.7%)	0.96
Gestational age at delivery, weeks	36.2 ± 1.28	35.3 ± 2.05	0.15
Gestational age at delivery < 36.0 weeks, n(%)	14 (33.3%)	15 (50%)	**0.04**
Gestational age at delivery < 34.0 weeks, n(%)	2 (4.8%)	8 (26.7%)	** < 0.001**
Intertwin birth interval, min	5.0 ± 6.10	2.0 ± 2.41	** < 0.001**
First twin birthweight, grams	2,413 ± 384.10	2,228 ± 456.68	0.07
Second twin birthweight, grams	2,385 ± 437.22	2,134 ± 447.62	**0.02**

*CS* cesarean section, *IVF* in vitro fertilization

### Obstetric outcomes in PVD and PCS groups

In the PVD group, 64.3% of women had vaginal delivery. Combined delivery (vaginal delivery of the first twin and intrapartum CS of the second twin) was performed in only one woman (2.4%). Fourteen cases (33.3%) from the PVD group were delivered via emergency CS because of non-reassuring fetal status (*n* = 7) or labor arrest (*n* = 7).

For pregnancies assigned to PVD group, 57.1% of pregnancies (24/42) had induction of labor. The main indication of labor induction was to avoid delivery above 37.6 weeks (22/24, 91.7%), followed by prenatal diagnosis of mild IUGR (2/24, 8.3%). 42.9% of pregnancies (18/42) had a spontaneous onset of labor. The rate of successful vaginal delivery was higher in the spontaneous onset of labor subgroup (72.2% vs. 58.3%, *p* = 0.35).

### Perinatal outcomes in PVD and PCS groups

Table 3 lists the incidence of adverse perinatal outcomes in each group. Composite perinatal morbidity was observed in at least one fetus in 3.6% of pregnancies (3/84) in the PVD group, and in 8.3% of pregnancies (5/60) in the PCS group [aOR 1.36 (95% CI 0.24–7.81), *P* = 0.22]. The rate of composite perinatal morbidity was higher in the PCS group for the second twin [5.0% vs. 2.4%, aOR 3.20 (95% CI 0.16–65.88)], yet these differences did not reach statistical significance (*P* = 0.16). Composite perinatal morbidity was not observed in the eight cases in which breech extraction of the second twin was performed.

**Table 3:** Perinatal outcomes and NDI according to the planned mode of delivery (intention to treat)

	**Overall** ***n* = 144**	**PVD group** ***n* = 84**	**PCS group** ***n* = 60**	***P*-value**	**OR (95% CI)**	**aOR (95% CI)**^**b**^
**Composite perinatal morbidity, n(%)**	8 (5.6)	3 (3.6)	5 (8.3)	0.22	2.46 (0.56–10.69)	1.36 (0.24–7.81)
First twin	4/77 (5.6)	2/42 (4.8)	2/30 (3.3)	0.73	1.43 (0.19–10.75)	0.77 (0.074–8.04)
Second twin	4/77 (5.6)	1/42 (2.4)	3/30 (5)	0.16	4.56 (0.45–46.11)	3.20 (0.16–65.88)
> 34 week’s	4/124 (3.2)	2/80 (2.5)	2/44 (4.5)	0.54	1.86 (0.25–13.66)	4.69 (0.85–25.81)
> 36 week’s	3/86 (3.5)	1/56 (1.8)	2/30 (6.7)	0.24	3.93 (0.34–45.22)	5.21 (0.49–55.63)
**5-min Apgar score < 4**	0	0	0	-	^a^	^a^
**5-min Apgar score < 7**	1 (0.7)	1 (1.2)	0	> 0.99	^a^	^a^
**Neonatal death**	0	0	0	-	^a^	^a^
**Respiratory morbidity (respiratory distress síndrome and/or bronchopulmonary dysplasia)**	0	0	0	-	^a^	^a^
**Intraventricular hemorrhage**	5 (3.5)	2 (2.4)	3 (5.0)	0.40	2.16 (0.35–13.33)	1.64 (0.19–14.57)
**Periventricular leukomalacia**	1 (0.7)	0	1 (1.7)	0.42	^a^	^a^
**Necrotizing enterocolitis**	1 (0.7)	0	1 (1.7)	0.42	^a^	^a^
**Sepsis**	0	0	0	-	^a^	^a^
**2-year-Neurodevelopmental impairment, n(%)**	10/140 (7.1)	4/81 (4.9)	6 / 59 (10.2)	0.26	2.11 (0.57–7.84)	1.53 (0.37–6.29)
Severe neurodevelopmental impairment	2/140 (1.4)	0	2 / 59 (3.4)	0.18	^a^	^a^

^a^OR cannot be calculated reliably due to zero events in at least one group

^b^Adjusted for birth weight (per gram) and gestational age (per day)

Due to the low frequency of the different items included in terms of composite perinatal morbidity, we did not find significant differences between the two groups in any of them. No cases resulted in neonatal death in either group.

As secondary outcome, we compared the outcomes respect to the onset of labor when a vaginal delivery was attempted (Table 4). The mean gestational age was significantly lower in the spontaneous delivery group as, following the Department protocol, before 35 weeks no labor induction is performed (35.4 vs. 36.6 weeks, *P* = 0.001). The rate of composite perinatal morbidity was higher in the spontaneous trial of labor subgroup (8.3% vs. 0%, *P* = 0.08), although the differences did not reach statistical significance. This is mainly related to an IVH event grade I occurred in both twins born from the same delivery at 34.2 weeks. This IVH event has no impact on 2-years neurodevelopment.

**Table 4:** Perinatal outcomes and NDI according to the onset of labor in PVD group

	**Overall** ***n* = 84**	**Induced onset of labor** ***n* = 48**	**Spontaneous onset of labor** ***n* = 36**	***P*-value**
**Gestational age at delivery, weeks (± SD)**	36.2 ± 1.28	36.6 ± 0.70	35.4 ± 1.39	**0.001**
**Composite perinatal morbidity, n(%)**	3 (3.6)	0	3 (8.3)	0.08
First twin	2/42 (4.8)	0	2/18 (11.1)	0.18
Second twin	1/42 (2.4)	0	1/18 (5.6)	0.43
> 34 week’s	2/80 (2.5)	0/48	2/32 (6.3)	0.16
> 36 week’s	1/56 (1.8)	0/42	1/14 (7.1)	0.25
**5-min Apgar score < 4**	0	0	0	-
**5-min Apgar score < 7**	1 (1.2)	0	1 (2.9)	0.43
**Neonatal death**	0	0	0	-
**Respiratory morbidity (respiratory distress síndrome and/or bronchopulmonary dysplasia)**	0	0	0	-
**Intraventricular hemorrhage**	2 (2.4)	0	2 (5.6)*	0.18
**Periventricular leukomalacia**	0	0	0	-
**Necrotizing enterocolitis**	0	0	0	-
**Sepsis**	0	0	0	-
**2-year-Neurodevelopmental impairment, n(%)**	4/81 (4.9)	0/47	4/34 (11.8)	**0.03**
Severe neurodevelopmental impairment	0/81	0/47	0/34	-

Finally, we performed a comparison between the perinatal outcomes of induced onset of labor group vs. PCS group (Table 5), being homogeneous groups in terms of gestational age at delivery and weight at birth. We found a higher composite perinatal morbidity in the PCS group, although without significant differences (8.3% vs. 0%, *P* = 0.06).

**Table 5:** Perinatal outcomes and NDI in PCS group compared to induced onset of labor subgroup

	**Overall** ***n* = 108**	**Induced onset of labor** ***n* = 48**	**PCS** ***n* = 60**	***P*-value**
**Composite perinatal morbidity, n(%)**	5	0	5 (8.3)	0.06
First twin	2/54	0	2/30 (3.3)	0.50
Second twin	3/54	0	3/30 (5)	0.25
> 34 week’s	2	0/48	2/44 (4.5)	0.23
> 36 week’s	2	0/42	2/30 (6.7)	0.17
**5-min Apgar score < 4**	0	0	0	-
**5-min Apgar score < 7**	0	0	0	-
**Neonatal death**	0	0	0	-
**Respiratory morbidity (respiratory distress síndrome and/or bronchopulmonary dysplasia)**	0	0	0	-
**Intraventricular hemorrhage**	3	0	3 (5.0)	0.25
**Periventricular leukomalacia**	1	0	1 (1.7)	> 0.99
**Necrotizing enterocolitis**	1	0	1 (1.7)	> 0.99
**Sepsis**	0	0	0	-
**2-year-Neurodevelopmental impairment, n(%)**	6/106 (5.7)	0 / 47	6/59 (10.2)	**0.03**
Severe neurodevelopmental impairment	2/106 (1.9)	0 / 47	2/59 (3.4)	0.50

### 2-year-neurodevelopment status

Finally, we studied the neurodevelopment of the children at 2-year-age (Tables 3, 4, 5). Neurodevelopment status data were available for 140 children of the initial cohort: 81 from the PVD group and 59 from the PCS group. Among these infants, NDI rate was slightly higher in the PCS group, without reaching statistical significance [10.2% vs. 4.9%, aOR 1.53 (95% CI 0.37–6.29), *P* = 0.26].

We found a lower NDI rate in the induced onset group compared to spontaneous onset of labor group (0% vs. 11.8%, *P* = 0.03), with no events of severe NDI. The rate of NDI was also lower in the induced onset of labor group compared to the PCS group (10.2% vs. 0%, *P* = 0.03), without reaching significant differences in the appearance of severe NDI events (3.4% vs. 0%, *P* = 0.50).

## Discussion

### Main findings

In this study, the results indicated that PVD is not associated with a high risk of adverse perinatal outcomes or NDI at 2 years in uncomplicated MCDA twins. Attempted vaginal delivery seems to be a safe option with a high vaginal delivery rate.

In the PCS group, the composite perinatal morbidity was higher (aOR 1.36, 95% CI 0.24–7.81), also finding a slightly higher rate of NDI at 2 years (10.2% vs. 4.9%) and severe NDI (3.4% vs. 0%).

Regarding the onset of labor in PVD group, induction of labor has been shown to be a safe option when performed above 35 gestational weeks. In our study, we have found a higher perinatal morbidity in the spontaneous trial of labor subgroup, with a higher rate of 2-year-NDI. Nevertheless, the composite perinatal morbidity in both subgroups was lower than in PCS group.

### Interpretation and comparison to current literature

MCDA twins are considered to be at highest risk of perinatal morbidity and mortality mainly related to acute hemodynamic intrapartum changes mediated by placental vascular anastomoses [11, 12]. In addition, despite the relevance of chorionicity, most studies addressing the contribution of the mode of delivery in perinatal morbidity have not been stratified accordingly.

To date, only eight studies have taken chorionicity into account when analyzing the optimal mode of delivery in uncomplicated twins, with different designs and neonatal morbidity criteria [17–24]. The most relevant data of each study are summarized in Table 6. None of them considered the mode of onset of labor nor long-term NDI when attempted vaginal delivery.

**Table 6:** Selective analysis of uncomplicated MCDA twin delivery in terms of composite perinatal morbidity

		**Design of study**	**Centers involved**	**GA**	**n**	**PVD**	**PCS**	**Difference induced labor from spontaneous onset**	**Items included**	**OR (CI 95%)**	***P*-value**	**Results**
*Sau *et al*. 2006 *[17]	Retrospective cohort study		Single-center	≥ 24 weeks	60	Not defined	Not defined	No	5-min Apgar score, umbilical pH, respiratory distress syndrome, mortality, ventilation	- (-)	(-)	Differences in composite morbidity not defined
*Hack *et al*. 2011 *[18]	Secondary analysis of a retrospective cohort study		Multicenter	≥ 32 weeks	902	752	150	No	5-min Apgar score, pH, respiratory distress syndrome, intrauterine fetal demise, mortality	2.0 (1.0–4.2)	(-)	No significant differences between: - PVD vs. PCS
*Hoffmann *et al*. 2012 *[19]	Retrospective cohort study		Multicenter	36 weeks	115	63	52	No	5-min Apgar score, pH, mortality	0.9 (0.3–3.0)	0.83	No significant differences between: - PVD vs. PCS
*Weisz *et al*. 2012 *[20]	Retrospective cohort study		Single-center	35 + 0–37 + 6 weeks	89	38	511	No (only in average of vaginal delivery, not morbidity)	5-min Apgar score, pH, respiratory distress syndrome, sepsis, mortality	- (-)	0.24	No significant differences between: - PVD vs. PCS
*Yamashita *et al*. 2014 *[21]	Retrospective cohort study		Single-center	≥ 36 weeks	295	187	108	No	5-min Apgar scores < 7, intrauterine fetal demise after 36 weeks’ gestation, neonatal death, umbilical artery pH < 7.1, hypoxic ischemic encephalopathy, meconium aspiration syndrome, respiratory distress syndrome, or acute feto-fetal hemorrhage	0.4 (0.1–2.0)	0.29	No differences between PVD vs. PCS (univariate analysis of perinatal factors for the composite adverse outcome, but no comparation between groups)
*Ylilehto *et al*. 2017 *[22]	Secondary analysis of a retrospective cohort study		Single-center	≥ 37 weeks	73	59	14	No	5-min Apgar < 4, 5-min Apgar < 7, umbilical artery pH < 7.05 and < 7.00, NICU admission	- (-)	> 0.99	No significant differences between: - PVD vs. PCS
*Ylilehto *et al*. 2020 *[23]	Secondary analysis of a retrospective cohort study		Single-center	Not specified to this cohort (primary cohort – mono and dichorionic: 32 + 0– 36 + 6 weeks of gestation)	55	40	15	No	5-min Apgar < 4, 5-min Apgar < 7, umbilical artery pH < 7.05, NICU admission, intracerebral hemorrhage gr. III-IV, respiratory morbidity, composite neonatal morbidity	- (-)	0.18	No significant differences between: - PVD vs. PCS
*Aviram *et al*. 2020 *[24]	Secondary analysis of a randomized, controlled trial (Twin Birth Study, Barret et al. 2013)		Multicenter	32 + 0– 37 + 6 weeks	670	324	346	No	5-min Apgar < 4, Stillborn, neonatal death, primary, umbilical artery pH < 7.0, NICU admission > 48 h, assisted ventilation, necrotizing enterocolitis, periventricular leukomalacia, composite outcome	- (-)	0.25	No significant differences between: - PVD vs. PCS - Twin A vs. Twin B - Monochorionic vs. Dichorionic

*PVD* planned vaginal delivery, *PCS* planned cesarean section

Our study shows that attempted vaginal delivery for uncomplicated MCDA twins is a safe management option, and has a low perinatal morbidity rate. These findings are consistent with those reported in the above-mentioned studies. Even though it is not a large sample, the data suggest that in uncomplicated MCDA pregnancies greater than 32.0 weeks of gestation, when first twin is in vertex presentation, PCS does not seem to avoid perinatal adverse events, but rather shows a slight increase in them (aOR 1.36, 95% CI 0.24–7.81).

The gestational age at delivery in pregnancies included in the different studies is highly variable, ranging from 24.0 to 38.6 weeks. In our study, we decided to include only moderate to late preterm births (32.0 – 36.6 weeks) to avoid masking a protective effect of cesarean section in that range of gestational age.

The impact of the mode of delivery in twins on 2-year-neurodevelopment was studied by Asztalos et al. in 2016 [28]. The authors performed a secondary analysis of the *Twin Birth Study*, a randomized controlled trial designed to compare planned vaginal delivery and PCS [5]. Using the Ages and Stages Questionnaire, the authors conclude that a policy based in PCS provides no benefit to children at 2 years of age [NDI 5.99% vs. 5.83%, OR 1.04 (95% CI 0.77–1.41), *P* = 0.79]. However, they do not stratify their data in relation to chorionicity. Our study, which takes chorionicity in consideration, yields similar results, showing a slightly higher NDI rate in PCS group. Furthermore, the rate of 2-year-NDI is similar to that reported by other authors when a specific analysis of MCDA pregnancies is performed [29].

### Strengths and limitations

The major strength of this study is that all pregnancies included were fully evaluated and delivered in a major single tertiary center, with a long tradition of vaginal delivery and breech extraction of the second twin. Therefore, the results can be extrapolated to centers that meet the same conditions.

To our knowledge, this is the first study considering the mode of onset of labor as well as 2-years-neurodevelopment after structured follow-up in a single institution. Thus, we have been able to define a more detailed and accurate perinatal outcome measures than using registry-based data, even though the study population is not large.

Our study has the limitations of retrospective studies. Also, the sample size may be small to detect low frequency adverse effects.

## Conclusion

The main aim of this study has been to provide evidence and support to parents and practitioners when deciding the mode of labor and delivery in uncomplicated MCDA twin gestations greater than 32 weeks.

In uncomplicated monochorionic diamniotic twins at ≥ 32 weeks of gestation, when first twin is in vertex presentation, attempt of vaginal delivery is a safe management option in terms of perinatal morbidity as well as long-term neurodevelopment, with a high vaginal delivery rate.

## Data Availability

The datasets used during the current study are available from the corresponding author on reasonable request.

## Abbreviations

aOR: Adjusted odds ratio
CS: Cesarean section
MCDA: Monochorionic diamniotic
NDI: Neurodevelopmental impairment
PCS: Planned cesarean section
PVD: Planned vaginal delivery
